# Calreticulin localizes to plant intra/extracellular peripheries of highly specialized cells involved in pollen-pistil interactions

**DOI:** 10.1007/s00709-017-1134-8

**Published:** 2017-06-15

**Authors:** Piotr Wasąg, Anna Suwińska, Przemysław Zakrzewski, Jakub Walczewski, Robert Lenartowski, Marta Lenartowska

**Affiliations:** 1 Laboratory of Isotope and Instrumental Analysis, Faculty of Biology and Environmental Protection, Nicolaus Copernicus University in Toruń, Toruń, Poland; 2 Laboratory of Developmental Biology, Faculty of Biology and Environmental Protection, Nicolaus Copernicus University in Toruń, Toruń, Poland; 3 Department of Plant Pathology, Plant Breeding and Acclimatization Institute, National Research Institute, Radzików, Poland

**Keywords:** Cell wall, Exchangeable Ca^2+^, Filiform apparatus, Plasmodesmata, Pollen tube, Style transmitting tissue

## Abstract

Calcium (Ca^2+^) plays essential roles in generative reproduction of angiosperms, but the sites and mechanisms of Ca^2+^ storage and mobilization during pollen-pistil interactions have not been fully defined. Both external and internal Ca^2+^ stores are likely important during male gametophyte communication with the sporophytic and gametophytic cells within the pistil. Given that calreticulin (CRT), a Ca^2+^-buffering protein, is able to bind Ca^2+^ reversibly, it can serve as a mobile store of easily releasable Ca^2+^ (so called an exchangeable Ca^2+^) in eukaryotic cells. CRT has typical endoplasmic reticulum (ER) targeting and retention signals and resides primarily in the ER. However, localization of this protein outside the ER has also been revealed in both animal and plant cells, including Golgi/dictyosomes, nucleus, plasma membrane/cell surface, plasmodesmata, and even extracellular matrix. These findings indicate that CRT may function in a variety of different cell compartments and specialized structures. We have recently shown that CRT is highly expressed and accumulated in the ER of plant cells involved in pollen-pistil interactions in *Petunia*, and we proposed an essential role for CRT in intracellular Ca^2+^ storage and mobilization during the key reproductive events. Here, we demonstrate that both CRT and exchangeable Ca^2+^ are localized in the intra/extracellular peripheries of highly specialized plant cells, such as the pistil transmitting tract cells, pollen tubes, nucellus cells surrounding the embryo sac, and synergids. Based on our present results, we propose that extracellularly located CRT is also involved in Ca^2+^ storage and mobilization during sexual reproduction of angiosperms.

## Introduction

CRT is a Ca^2+^-binding/buffering protein implicated in many cellular functions, including lectin-like chaperoning, Ca^2+^ storage and signaling, regulation of gene expression, cell adhesion, regeneration, immunity, and apoptosis (see reviews by Michalak et al. 2009; Jia et al. 2009; Thelin et al. 2011). This multifunctional protein promotes folding and quality control of newly synthesized glycoproteins in the ER via the CRT/calnexin cycle. CRT is also involved in ER Ca^2+^ capacity and thus stabilization of Ca^2+^ homoeostasis in the cell cytoplasm. Since CRT has typical ER targeting and retention signals, it resides primarily in the ER lumen. However, localization of this protein outside the ER has also been observed in eukaryotic cells. For example in animals, CRT was detected in the nucleus, nuclear matrix, on the surface of mitotic chromosomes, and in the extracellular matrix (see review by Michalak et al. 2009). Plant CRT was found in many different compartments and structures, including dictyosomes/vesicles (Borisjuk et al. 1998; Navazio et al. 2002; Lenartowska et al. 2002, 2009; Hsieh and Huang 2005; Nardi et al. 2006; Lenartowski et al. 2015; Niedojadło et al. 2015), the cytosol (Lenartowska et al. 2002; Jia et al. 2008), protein bodies (Torres et al. 2001; Šamaj et al. 2008), nucleus (Denecke et al. 1995; Napier et al. 1995; Lenartowska et al. 2002; Lenartowski et al. 2015), plasma membrane/cell surface (Borisjuk et al. 1998; Lenartowska et al. 2002; Navazio et al. 2002; Šamaj et al. 2008), plasmodesmata (Baluška et al. 1999; Laporte et al. 2003; Bayer et al. 2004; Chen et al. 2005; Lenartowska et al. 2009; Bilska and Sowiński 2010; Christensen et al. 2010), and the cell wall (Lenartowska et al. 2002, 2009; Lenartowski et al. 2015; Luczak et al. 2015; Niedojadło et al. 2015). These varied locations suggest that CRT may function in different plant cell compartments and specialized structures, including extracellular regions.

Sexual reproduction of angiosperms involves complicated pollen-pistil interactions during directional growth of pollen tubes through the pistil transmitting tract, pollen tube entry to the ovule, and then the male and female gametophytes communication. Ca^2+^ has long been recognized to play essential signaling, physiological, and regulatory roles in these reproductive events (see reviews by Ge et al. 2007; Dresselhaus and Franklin-Tong 2013; Steinhorst and Kudla 2013). Given that CRT is able to bind Ca^2+^ reversibly (exchangeable Ca^2+^), its Ca^2+^-buffering activity has the potential to be involved in modulation of Ca^2+^ concentrations in the ER and consequently in the cytosol. Recent work from our lab provided evidence that CRT is highly expressed and accumulated in the ER of *Petunia* cells during pistil transmitting tract maturation, the progamic phase and early embryogenesis (Lenartowski et al. 2014, 2015). Since the ER cisternae and Golgi stacks are known to be the effective Ca^2+^ stores in eukaryotic cells (see review by Vandecaetsbeek et al. 2011), we proposed an essential role for CRT in intracellular Ca^2+^ storage and mobilization during these key reproductive events (Lenartowski et al. 2014, 2015). We have also found that CRT is located on the cell membrane/surface and in the apoplast of highly specialized plant cells involved in pollen-pistil interactions (Lenartowska et al. 2002, 2009; Lenartowski et al. 2015). Localization of CRT outside the protoplast in different plant cells has been also confirmed by other authors (Borisjuk et al. 1998; Navazio et al. 2002; Šamaj et al. 2008; Luczak et al. 2015; Niedojadło et al. 2015). Since both internal and external Ca^2+^ stores are likely important during communication of the male gametophyte and the female sporophyte/gametophyte cells (see reviews by Ge et al. 2007; Dresselhaus and Franklin-Tong 2013; Steinhorst and Kudla 2013), in this report we focus on CRT located in intra/extracellular peripheries in the context of its probable role/s in mobile Ca^2+^ storage during pollen-pistil interactions in angiosperms.

## Materials and methods

### Plant material

Commercial cultivars of *Petunia hybrida* and *Haemanthus albiflos* were grown at room temperature, and whole pistils were dissected from unpollinated and pollinated flowers. Semithin sections of styles (*Petunia*) and ovules (*Petunia* and *Haemanthus*) were prepared according to standard protocols, stained with 0.1% methylene blue and observed by light microscopy. Then, selected tissue samples of styles and ovules were prepared for immunocytochemical and cytochemical (potassium antimonate precipitation) studies according to the protocols as described below. All experiments were repeated many times during several growing seasons with similar results.

### Immunocytochemical studies

Immunofluorescence and immunogold localizations of CRT were performed according to protocols described previously (Lenartowska et al. 2009; Lenartowski et al. 2015). In brief, samples of styles and ovules were fixed with 4% (*v*/*v*) formaldehyde and 0.25% (*v*/*v*) glutaraldehyde in phosphate-buffered saline (PBS, pH 7.2) for 1 h at room temperature (slight vacuum infiltration) followed by overnight fixation at 4 °C. Fixed samples were dehydrated in graduated ethanol concentrations, embedded in LR Gold resin (Fluka) according to the standard protocol, and then semithin and ultrathin longitudinal or cross-sections were collected on microscope slides covered with Biobond (BBInternational) or Formvar-coated nickel grids. After blocking in 3–5% bovine serum albumin (Sigma-Aldrich), sections were incubated with a rabbit polyclonal antibody against maize CRT (CRT PAb, Napier et al. 1995) and then with goat anti-rabbit IgG Cy3^®^ secondary antibody (Sigma-Aldrich) or with 10–20 nm diameter gold-conjugated goat anti-rabbit IgG antibody (BBInternational). As a final step, DNA was stained with 2 μg/ml 4′, 6-diamidino2-phenylindole (DAPI, Fluka). In the controls, incubations with the CRT PAb were omitted. Images were acquired using an Olympus BX50 fluorescence microscope, Olympus Xc50 digital color camera, and CellB software (Olympus Soft Imaging Solutions gmbH); ultrathin sections were examined by transmission electron microscopy (Jeol EM 1010) at 80 kV.

For CRT and callose immunolocalizations, a double-labeling technique was performed as previously described (Lenartowska et al. 2009). After blocking with 5% BSA (Sigma-Aldrich), ultrathin sections were treated with two kinds of primary antibodies: CRT PAb and monoclonal anti-(1 → 3)-*β*-glucan antibody (Cal MAb, Biosupplies). Signals were detected with the following secondary antibodies: 20 nm diameter gold-conjugated goat anti-rabbit IgG (for CRT) and 10 nm diameter gold-conjugated goat anti-mouse IgG (for callose), both from BBInternational. To quantitate the amount of CRT localized intra/extracellularly in different *Petunia* cells involved in pollen-pistil interactions, the average number of gold traces was determined in different cell sections of compartments/structures (min. 20) labeled with CRT PAb conjugated with immunogold secondary antibody. In the negative control, incubation with the primary antibodies was omitted. To verify if the CRT PAb specifically bound to protein epitopes, the immunolocalizaton was performed on ultrathin sections pretreated by incubation with a proteinase K solution (Lenartowska et al. 2002). Finally, the sections were stained with 2.5% (*w*/*v*) uranyl acetate and 0.4% (*w*/*v*) lead citrate solutions and examined by transmission electron microscopy as above. The specificity of maize CRT PAb in *Petunia* and *Haemanthus* was previously verified by immunoblotting (Lenartowska et al. 2009; Lenartowski et al. 2015).

### Visualization of loosely bound Ca^2+^ by potassium antimonate precipitation

Localization of exchangeable Ca^2+^ was performed according to the protocol described previously (Lenartowska et al. 2009; Lenartowski et al. 2015). In brief, samples of styles and ovules dissected from unpollinated/pollinated pistils were fixed with freshly prepared 2% (*w*/*v*) potassium antimonate, 2% (*v*/*v*) glutaraldehyde, and 2% (*v*/*v*) formaldehyde in 0.1 M phosphate buffer (KH_2_PO_4_, pH 7.8) for 4 h at room temperature, and then subsequently postfixed with 1% (*v*/*v*) osmium tetroxide (OsO_4_) in the same buffer-antimonate solution for 12 h at 4 °C. Next, samples were dehydrated in graduated ethanol concentrations and embedded in Poly/Bed 812 resin (Polysciences) according to the standard protocol. Ultrathin longitudinal or cross-sections were collected on copper grids, stained with 2.5% (*w*/*v*) uranyl acetate and 0.4% (*w*/*v*) lead citrate solutions, and examined by transmission electron microscopy as above. The presence of Ca^2+^ in the precipitates was confirmed previously using energy-dispersive X-ray microanalysis (Bednarska et al. 2005).

## Results

### CRT is present in intra/extracellular peripheries of the stylar transmitting tissue and pollen tubes

**Fig. 1:**
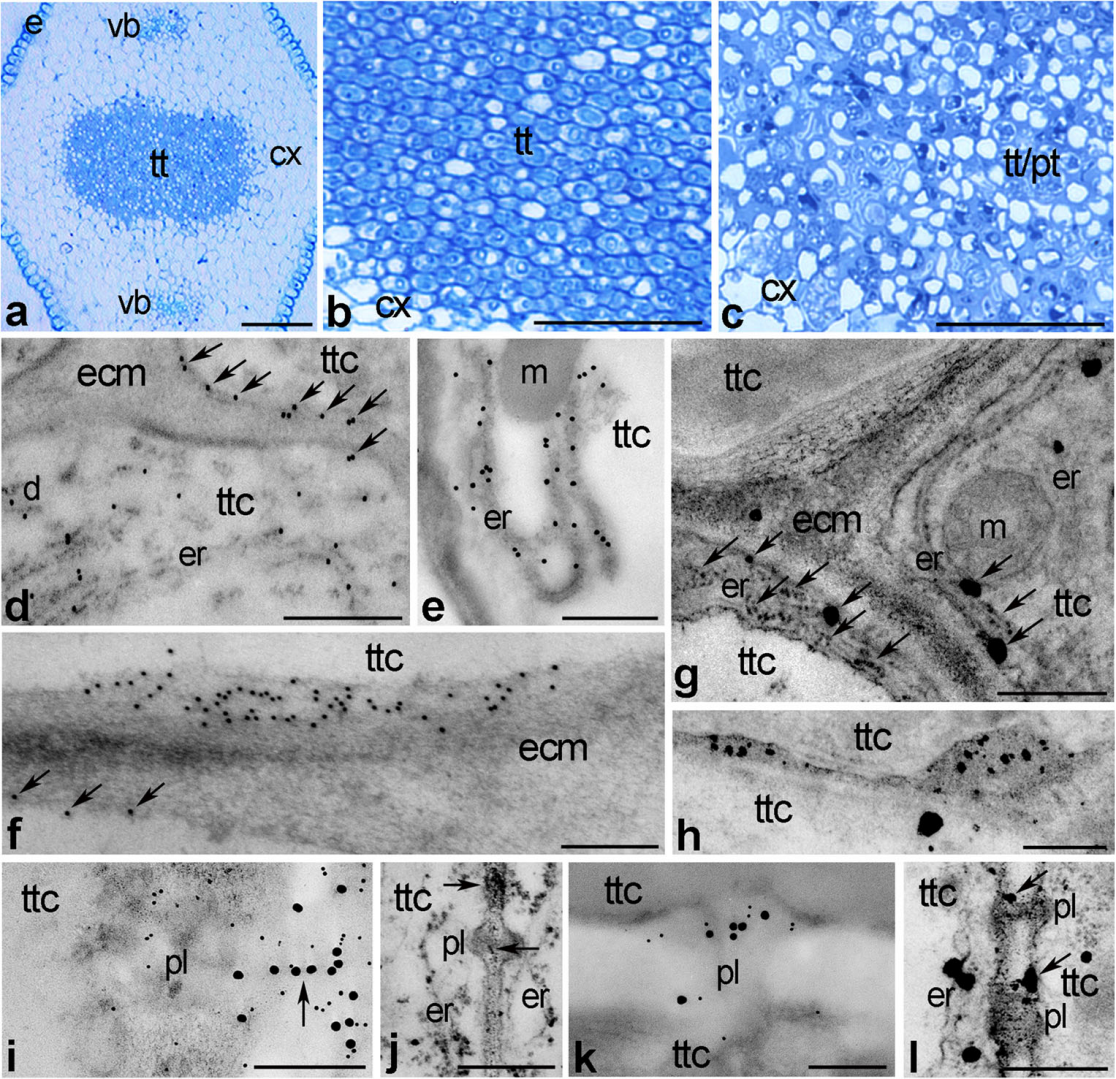
Immunogold localization of CRT (**d**–**f**, **i**, **k**) and distribution of exchangeable Ca^2+^ (**g**, **h**, **j**, **l**) in *Petunia* transmitting cells. **a**–**c** Methylene *blue* stained cross-sections of the pistil style showing transmitting tissue before (**a**, **b**) and after pollination (**c**). **d**, **g**, **i**, **j** Distributions of CRT and loosely bound Ca^2+^ in transmitting cells before pollination. **e**, **f**, **h**, **k**, **l** Distributions of CRT and loosely-bound Ca^2+^ in transmitting cells after pollination. *cx* cortex, *d* dictyosome, *ecm* extracellular matrix, *er* endoplasmic reticulum, *m* mitochondria, *pl* plasmodesmata, *tt* transmitting tissue, *ttc* transmitting tissue cells, *pt* pollen tube, *vb* vascular bundle. *Bars* 50 μm (**a**–**c**), 500 nm (**d**, **e**, **g**, **j**, **l**), 200 nm (**f**, **h**, **i**, **k**)

We first wished to determine if CRT is extracellularly localized in the stylar transmitting tract linking the stigma with the ovary. To investigate this, samples of unpollinated and pollinated *Petunia* styles were processed for immunogold labeling and visualized by electron microscopy. As shown in semithin sections stained with methylene blue, *Petunia* has a solid style with highly specialized transmitting tissue composed of secretory cells (Fig. 1a, b). The extracellular matrix of this tissue is enriched with exudates and forms the appropriate physical and nutritional medium for pollen tube growth in vivo (Fig. 1c).

Within the cytoplasm of transmitting cells, CRT was typically localized in the ER, both in unpollinated and pollinated pistils (Fig. 1d, e, respectively). However, before pollination, numerous gold traces were also detected along the edge of these cells, on the border between the protoplast and the cell wall (Fig. 1d, arrows). After pollination, CRT was frequently observed at the cellular peripheries (Fig. 1f, arrows) and accumulated in the plasma membrane-attached patches (Fig. 1f). Consistent with CRT being a Ca^2+^-binding/buffering protein, Ca^2+^-antimonate precipitates (Ca^2+^ ppts corresponding to exchangeable Ca^2+^) were observed in the same localizations where CRT was found; there were the ER (Fig. 1g) and several patches adjacent to the cell wall of the transmitting cells (Fig. 1h). It should be noted that Ca^2+^ ppts were predominantly observed in the ER enriched peripheral cytoplasm (Fig. 1g, arrows). Epitopes binding CRT PAb were also found at plasmodesmata connecting transmitting cells. The specific linear pattern of the labeling is likely to correspond to ER in the cytoplasmic sleeve - an essential component of these narrow channels (Fig. 1i, arrow). Moreover, double-labeling experiments using both CRT PAb and Cal MAb clearly showed that CRT co-localized tightly with callose at the neck region of the plasmodesmata (Fig. 1i, k). Numerous Ca^2+^ ppts were found in the cortical ER attached to plasmodesmata (Fig. 1j, l) as well as with their central cavity and neck regions (Fig. 1j, l, arrows).

**Fig. 2:**
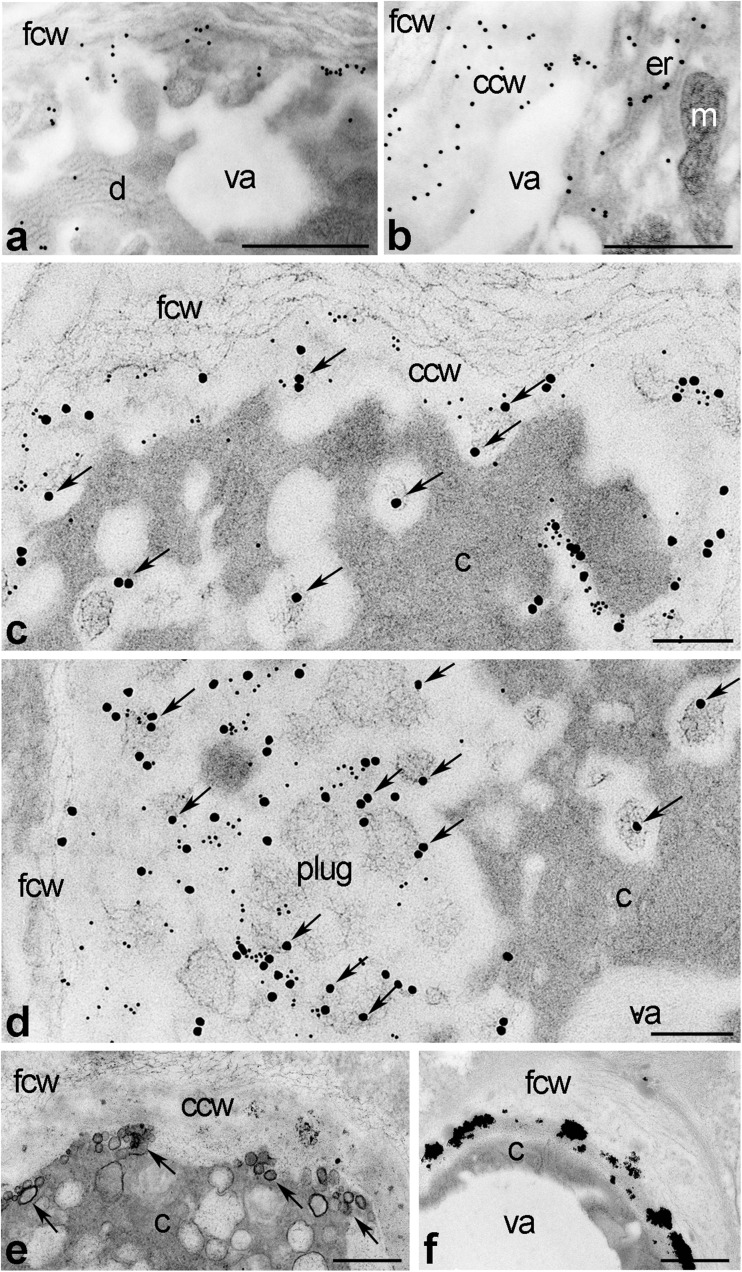
Immunogold localization of CRT (**a**–**d**) and visualization of exchangeable Ca^2+^ (**e**, **f**) in *Petunia* pollen tubes growing in situ. *c* cytoplasm, *ccw* callosic cell wall, *d* dictyosome, *e* endoplasmic reticulum, *fcw* fibrillar cell wal, *m* mitochondria, *plug* callose plug, *va* vacuole. *Bars* 1 μm (**e**, **f**), 500 nm (**a**, **b**), 200 nm (**c**, **d**)

After pollination, CRT labeling was confirmed in pollen tubes growing between the transmitting cells. Intracellularly, the protein was localized to the most prominent organelles in the pollen tube subapical zone, such as dictyosomes (Fig. 2a) and ER (Fig. 2b). Some gold traces were also found in the peripheral cytoplasm of the tube adjacent to the cell wall (Fig. 2a). Such CRT labeling often corresponded with the position of the ER within the cytoplasm (Fig. 2b). However, epitopes binding CRT PAb were also identified in the inner cell wall of the pollen tube (Fig. 2b). Double staining demonstrated that, similar to plasmodesmata, peripheral and extracellular CRT localizations in elongated pollen tubes were tightly correlated with callose deposition (Fig. 2c) and plug formation in the shank of highly elongated tube (Fig. 2d). It should be noted that CRT labeling in the inner callose wall was predominantly associated with several vesicles containing electron-dense cores; they were present in both the tube cytoplasm and the callose depositions (Fig. 2c, d, arrows). In contrast, the fibrillar outer cell wall of the pollen tube was devoid of CRT PAb and Cal MAb labeling (Fig. 2a–d). As we expected, numerous Ca^2+^ ppts were found in cellular peripheries and the extracellular space of elongated pollen tubes, including vesicles undergoing exocytosis (Fig. 2e, arrows) and the callosic cell wall of the tube (Fig. 2e, f).

## Both nucellus cell peripheries and filiform apparatus of the synergid accumulate CRT

**Fig. 3:**
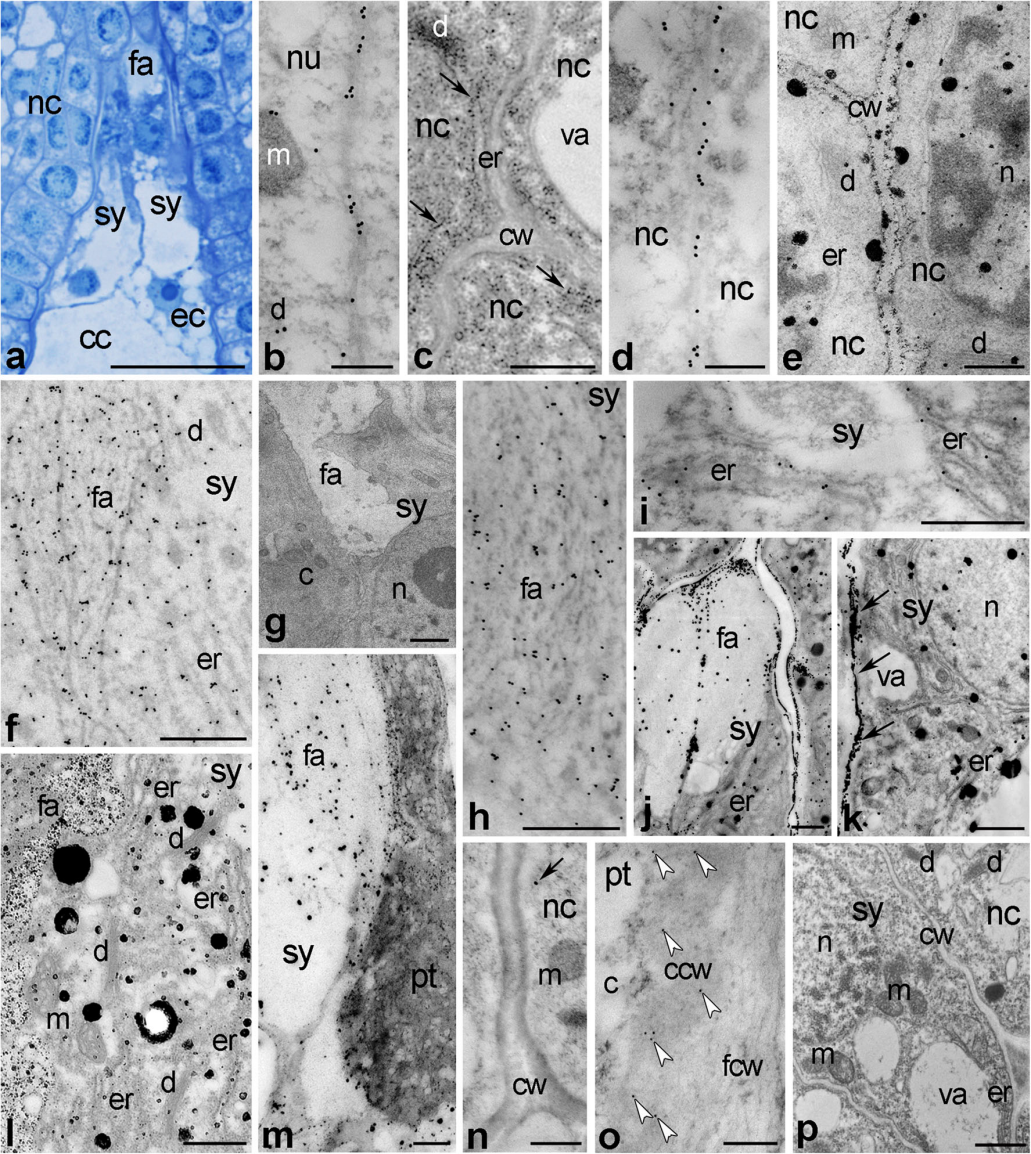
Immunogold localization of CRT (**b**, **d**, **f**, **h**, **i**, **m**) and distribution of exchangeable Ca^2+^ (**c**, **e**, **g**, **j**-**m**) in *Petunia* micropylar pole of the ovule. **a** Methylene *blue* stained longitudinal section of the embryo sac micropylar pole before pollination. **b**, **c**, **f**, **g**, **j**, **k** Distributions of CRT and loosely-bound Ca^2+^ in the micropylar pole of the ovule form unpollinated pistil. **d**, **e**, **h**, **i**, **l**, **m** Distributions of CRT and loosely–bound Ca^2+^ in the micropylar pole of the ovule form pollinated pistil. **n** Negative immunocytochemical control. **o** Proteinase K control. **p** Potassium antimonate precipitation control. *c* cytoplasm, *ccw* callosic cell wall, *cc* central cell, *cw* cell wall, *d* dictyosome, *ec* egg cell, *er* endoplasmic reticulum, *fa* filiform apparatus, *fcw* fibrillar cell wal, *m* mitochondria, *n* nucleus, *nc* nucellus, *pt* pollen tube, *sy* synergid, *va* vacuole. *Bars* 25 μm (**a**), 1 μm (**j**, **k**, **m**, **p**), 500 nm (**c**, **e**, **f**–**i**, **l**), 250 nm (**b**, **d**, **n**, **o**)

**Fig. 4:**
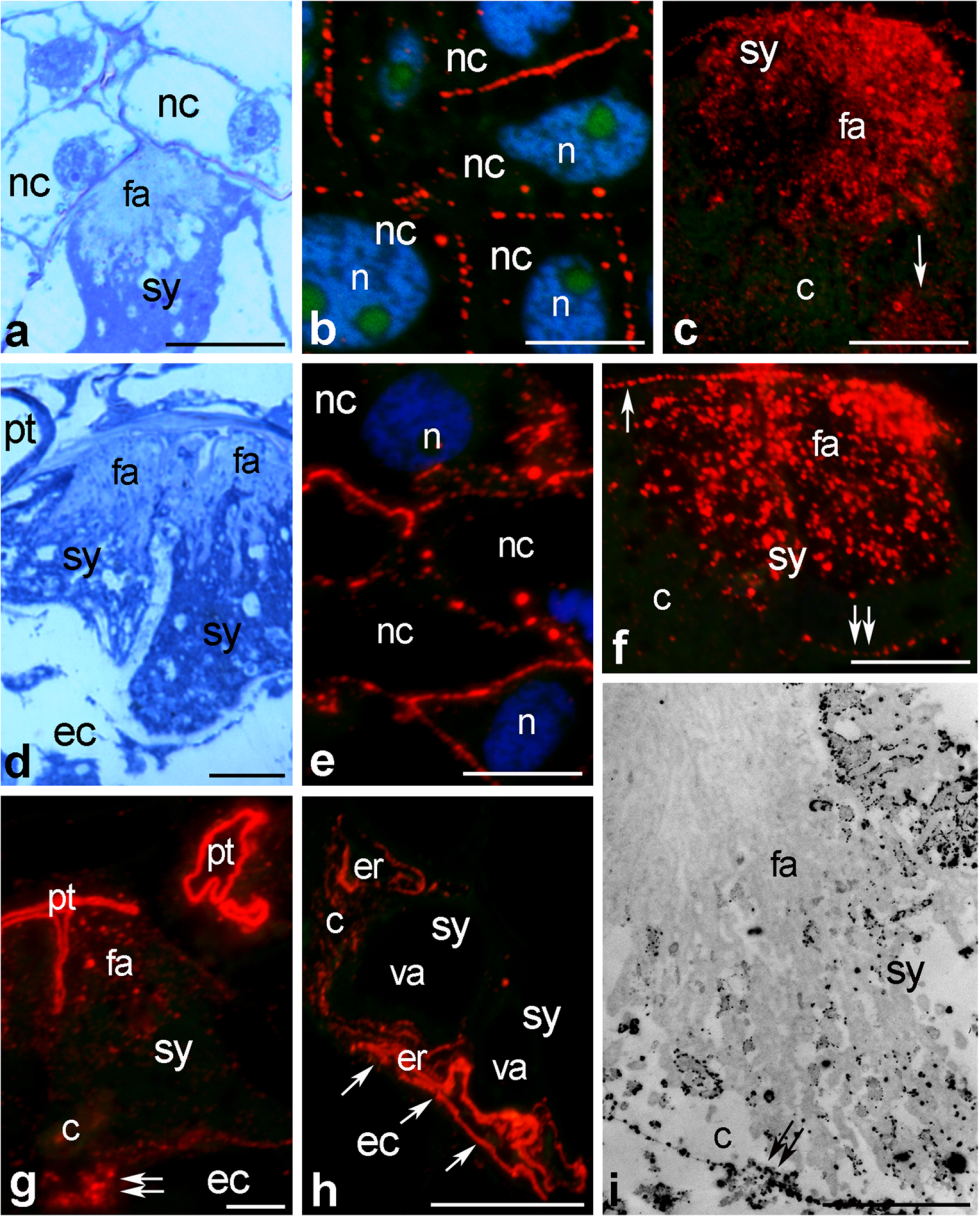
Immunofluorescence localization of CRT (**b**, **c**, **e**–**h**) in *Haemanthus* micropylar pole of the ovule and visualization of exchangeable Ca^2+^ (**i**) in *Haemanthus* filiform apparatus of the synergid. **a**, **d** Methylene *blue* stained longitudinal section of the embryo sac micropylar pole before (**a**) and after (**d**) pollination. *c* cytoplasm, *ec* egg cell, *er* endoplasmic reticulum, *fa* filiform apparatus, *n* nucleus, *nc* nucellus, *pt* pollen tube, *sy* synergid. *Bars* 20 μm (**b**, **c**, **e**, **f**), 10 μm (**a**, **d**, **g**–**i**)

Based on the results obtained for *Petunia* stylar transmitting tract, we hypothesized that CRT would be also a component of the extracellular space in the ovule. To test this idea, longitudinal sections through the ovules dissected from unpollinated/pollinated *Petunia* and *Haemanthus* pistils were prepared and processed for immunogold or immunofluorescence labeling. We prepared ultrathin or semithin sections because of different size of the ovules—small in *Petunia* and much bigger in *Haemanthus*. Dissected ovules were also fixed for visualization of exchangeable Ca^2+^. As shown in semithin sections stained with methylene blue, the synergid cell wall forms a highly thickened structure called the filiform apparatus at the micropylar end, consisting of numerous finger-like projections into the cytoplasm between sister synergids (Figs. 3a and 4a, d). This highly specialized structure was clearly visible before pollination and well-preserved during the progamic phase (Fig. 4a, d, respectively).

In *Petunia* ovules, the presence of CRT in intra/extracellular peripheries has been confirmed for the nucellus surrounding the embryo sac (Fig. 3b, d) and for the synergid cell (3f, h). Although it was difficult to discern whether CRT labeling in nucellus cells is limited to the protoplast/cell membrane adjacent to the cell wall or occurs in the apoplast, the localization pattern of CRT was comparable before and after pollination (Fig. 3b, d, respectively). An extremely high level of CRT was found in the synergid filiform apparatus where gold traces were uniformly distributed along the electron-dense fibrils (Fig. 3f, h). Intracellularly, CRT was found in the ER-rich cytoplasm of this cell (Fig. 3f, i). Such intense CRT PAb labeling of the synergid was observed both before and after pollination. In contrast, we found clear differences in the level, size and localization of Ca^2+^ ppts in both the nucellus and the synergid cells between the unpollinated and pollinated pistils. Before pollination, numerous but minor Ca^2+^ ppts were detected in the nucellus. They were localized mainly in the cortical ER (Fig. 3c, arrows) and dictyosomes (Fig. 3c), while the apoplast was devoid of ppts. After pollination, Ca^2+^ ppts strongly labeled the cell walls and several cell membrane-associated patches (Fig. 3e). The labeling was also found intracellularly, in dictyosomes and nuclei with associated ER (Fig. 3e). Similar to the nucellus, we did not find accumulation of Ca^2+^ ppts within the synergid before pollination (Fig. 3g). However, a drastic increase of exchangeable Ca^2+^ was observed in the synergid during the progamic phase (Fig. 3j, l, m). Extracellularly, Ca^2+^ ppts were evident in the filiform apparatus (Fig. 3j–m) along which the pollen tube elongated (Fig. 3m), and in the cell wall separating the embryo sac from the nucellus (Fig. 3k, arrows). Ca^2+^ labeling was also prominent in the synergid cell cytoplasm, including the ER (Fig. 3j–l), dictyosomes (Fig. 3l), and the nucleus (Fig. 3k). Since the presence of CRT in the extracellular space is somewhat controversial, the specificity of immunogold reaction was verified in two ways. First, no background was observed when control sections were incubated with only secondary gold-labeled antibodies; only single gold traces were found in the negative control (Fig. 3n, arrow). Second, binding of the CRT PAb to the cell wall was inhibited when the sections were pretreated with proteinase K to digest some protein epitopes (Fig. 3o). In this case, only Cal MAb labeling was preserved in the inner cell wall of the pollen tube (Fig. 3o, arrowheads). Thus, presence of CRT PAb within the pollen tube callose wall appears to be specific. The same control results were observed in the filiform apparatus (data not showed). Furthermore, a negative control for potassium antimonate precipitation showed Ca^2+^ ppts were absent in analyzed cells (Fig. 3p).

Finally, we investigated *Haemanthus* ovules processed for immunofluorescence labeling. We confirmed presence of CRT in the cytoplasm of nucellus cells and between these cells both before and after pollination (Fig. 4b, e, respectively). In the synergid, the strongest signals detected were associated with the filiform apparatus (Fig. 4c, f), the cell wall bordering tween synergids (Fig. 4f, arrow and double arrow), and the cytoplasm (Fig. 4c, arrow). During the late progamic phase, when pollen tubes penetrated the micropylar pole of the embryo sac, enrichment of CRT was still observed in the filiform apparatus (Fig. 4g) and in the cortical cytoplasm of the synergid (Fig. 4g, double arrow). This cytoplasmic region was extremely rich in ER strongly labeled by the CRT PAb (Fig. 4h, arrows). To obtain higher-resolution visualization of Ca^2+^ ppts in the synergid cell, we performed electron microscopy of ultrathin sections prepared for potassium antimonate precipitation. As we expected, a large mass of Ca^2+^ ppts was observed occupying the filiform apparatus (Fig. 4i) and the synergid cytoplasm (Fig. 4i, double arrow). Thus, we conclude that similar patterns of CRT and exchangeable Ca^2+^ localizations are present in the micropylar pole of the ovules dissected from *Petunia* and *Haemanthus* ovaries.

## Discussion

Here, we have demonstrated that both CRT and exchangeable Ca^2+^ are localized to the intra/extracellular peripheries of highly specialized plant cells, such as transmitting cells, pollen tubes, nucellus cells surrounding the embryo sac, and synergids. Although, some previous work from our lab and others indicated location of CRT in several cell wall patches, at the cell surface and in the cell wall of various cell types, these findings were somewhat controversial (Borisjuk et al. 1998; Lenartowska et al. 2002, 2009; Šamaj et al. 2008; Lenartowski et al. 2015; Niedojadło et al. 2015). However, recent elegant work of Luczak et al. (2015) provided clear evidence that CRT, similar to several other proteins, is always present in the cell walls of few plant species including maize, *Lupinus* and *Arabidopsis*. These results prompted us to perform a detailed analysis of CRT and exchangeable Ca^2+^ distributions in cellular peripheries and in the apoplast during pollen-pistil interactions. Our observations were supplemented by simple quantitative analysis of immunogold particles (summarized in Table 1) on the light of exchangeable Ca^2+^ distribution in investigated cells. To the best of our knowledge, this is the first study focused on extracellularly located CRT in context its probable role in mobile Ca^2+^ storage during sexual reproduction in angiosperms.

**Table 1:** The estimated amount of CRT localized intra/extracellularly in different *Petunia* cells and their organelles or structures

Localization	Amount high +++	Amount medium ++	Amount Low +
Transmitting cells	Cell wall patches	ER Cellular periphery	Cytoplasm (including dictyosomes) Plasmodesmata
Pollen tubes	Callosic wall/plugs	ER Cellular periphery	Cytoplasm (including dictyosomes) Dense-core vesicles
Nucellus cells	–	ER Cellular/extracellular peripheries	Cytoplasm (including dictyosomes)
Synergids	Filiform apparatus	ER	Cytoplasm (including dictyosomes)

### CRT and exchangeable Ca^2+^ are localized to intra/extracellular peripheries in the pistil transmitting tissue before and after pollination

We have demonstrated that CRT and exchangeable Ca^2+^ are typically located in the ER of transmitting cells, both in unpollinated and pollinated *Petunia* pistils. However, numerous gold traces (corresponding to CRT), as well as Ca^2+^ ppts (corresponding to exchangeable Ca^2+^ potentially bound by CRT), were also found in the cortical cytoplasm, the plasma membrane/wall attached patches and plasmodesmata. It should be noted that long ER cisternae commonly occurred in the peripheral cytoplasm of transmitting cells and were associated with intercellular connections. It has been also long recognized that plasmodesmata of higher plants contain a central strand of tightly compressed ER, called the rod or desmotubule, that creates the cytosolic sleeve providing continuity of cytoplasm between adjacent cells (see review by Kitagawa and Jackson 2017). Thus, localization of CRT in the cortical cytoplasm and plasmodesmata most likely corresponds with the ER. However, our double labeling experiments clearly showed that CRT co-localized also with callose at the neck region of plasmodesmata in *Petunia* transmitting cells, as we previously revealed for *Haemanthus* transmitting tract epidermis cells (Lenartowska et al. 2009). CRT was frequently shown to localize to plasmodesmta, though not all them accumulate this protein (Baluška et al. 2003; Laporte et al. 2003; Bayer et al. 2004; Chen et al. 2005; Lenartowska et al. 2009; Bilska and Sowiński 2010; Christensen et al. 2010; Demchenko et al. 2014). Furthermore, a comparison of the immunolocalization of CRT and callose in these structures favors a location of CRT in the ER. Baluška et al. (1999, 2003) originally suggested that CRT within plasmodesmata could gate their permeability via modulation of local/actual Ca^2+^ level as both CRT and plant-specific myosin VIII (likely regulated by Ca^2+^) are enriched at sink plasmodesmata. However, CRT was also found to be strongly expressed and accumulated near the neck region of closed channels in response to different stresses, irrespective of callose deposition (Bilska and Sowiński 2010; Demchenko et al. 2014). It was also shown that plasmodesmata connecting mature infected cells (in contrast to connecting uninfected or young infected cells) did not accumulate CRT or callose (Demchenko et al. 2014). These authors speculated that loss of callose and presumably also desmotubules leads to plasmodesmata becoming open channels and improves metabolite exchange between adjacent cells. Their observations led to hypothesis that CRT represents a universal mediator of fast plasmodesmata closure that plays a key role in cell-to-cell transport, as it was originally suggested (Baluška et al. 1999, 2003). In the light of these results, we argue that CRT localized within the plasmodesmata of transmitting cells regulates the architecture of intercellular connections via its Ca^2+^-binding/buffering capacity. Therefore, this protein seems to be involved in cell-to-cell communications within the style transmitting tract that intensify during the progamic phase.

In contrast, we did not find ER cisternae in the cell membrane-attached patches of transmitting cells, where both CRT and exchangeable Ca^2+^ were particularly concentrated after pollination. This observation suggests that CRT accumulation in cellular peripheries outside the ER, in several patches adjacent to the cell wall, is induced by pollination. Our findings are consistent with only one report of such CRT-rich patches in dividing *Nicotiana* protoplasts which exhibit high metabolic activity required for cell proliferation and the cell wall biosynthesis (Borisjuk et al. 1998). These authors speculated that CRT localized to cellular peripheries may function indirectly in signal perception (as an effective Ca^2+^ store), and/or in cell adhesion during the cell shape formation. Directional growth of the pollen tube in vivo involves both the tube cell adhesion to the style transmitting tract and diverse cell signaling pathways that regulate complicated pollen-pistil interactions (see review by Dresselhaus and Franklin-Tong 2013). Therefore, we suggest that CRT located at the cellular peripheries of the style transmitting cells may function as a mobile Ca^2+^ store involved in these Ca^2+^-dependent cellular processes during the progamic phase.

After pollination, CRT labeling was confirmed in pollen tubes that elongated within the extracellular matrix of *Petunia* stylar transmitting tract. The protein was identified in the peripheral ER-rich cytoplasm of the tube adjacent to the callosic wall and in this inner cell wall. In this region, CRT was usually associated with vesicles containing electron-dense cores in the tube cytoplasm and the callosic cell wall. As expected, numerous Ca^2+^ ppts were found in intra/extracellular peripheries of elongated *Petunia* pollen tubes, including vesicles undergoing exocytosis and the calosic cell wall of the tube. Similar results we found previously in *Haemanthus* pollen tubes (Lenartowska et al. 2009). Thus, we argue that localization pattern of CRT in the inner cell wall is universal in pollen tubes elongating in anatomically different pistil styles (solid in *Petunia* and hollow in *Haemantus*). Other studies have demonstrated that a lily pollen-specific protein LP2 and two pistil-specific proteins, 120 kDa and PELPIII glycoproteins, were also located in the callosic cell wall of developing pollen and pollen tubes growing in situ (Lind et al. 1996; Mogami et al. 2002; Graaf et al. 2003). In the light of these reports and based on the controls presented here, CRT labeling in the callose depositions is not an artifact. Despite these findings, it still remains unclear how CRT could leave the ER and move into the cell wall. The most likely explanations include existence of splice variants that can localize to different cell compartments, post-trancriptional modifications such as glycosylation, enzymatic modification of the glycan complexity, or degradation of the ER-retention signal (see reviews by Johnson et al. 2001; Michalak et al. 2009). Therefore, it is possible that CRT is translocated from the ER/dictyosomes to the cell periphery and then to the inner cell wall where it may play a role in external Ca^2+^ storage of the elongatinging pollen tube. Although the precise mechanism is still unclear, stabilization of a tip-focused Ca^2+^ gradient is critical for pollen germination and pollen tube growth (see reviews by Hepler et al. 2012; Steinhorst and Kudla 2013). We previously showed that in *Petunia* pollen tubes growing in vitro, CRT is translated on ER membrane-bound ribosomes and accumulated in the ER at the subapical zone of the tube, where plays a role in stabilizing Ca^2+^ homeostasis that is required for actomyosin-dependent cytoplasmic streaming, organelle positioning, vesicle trafficking, and cell wall biogenesis (Suwińska et al. 2015, 2017). Thus, we concluded that internal Ca^2+^ stores involving CRT activity are crucial for proper pollen tube elongation. Based on our present data showing both CRT and exchangeable Ca^2+^ in the callosic cell wall of the pollen tube, we cannot exclude the possibility that this external Ca^2+^ store is equally important in polar tip growth of the tube. It has been long suggested that both the concentration/availability of Ca^2+^ and the degree of pectin esterification are crucial for the cell wall expansion and the pollen tube tip growth (see review by Hepler et al. 2013). However, the neutral polymer of 1,3-β-glucan does not bind Ca^2+^. Thus, an attractive hypothesis suggests that excess of Ca^2+^ is translocated from the pollen tube tip cytosol not only to the ER but also to the callosic cell wall, and bound by CRT present there in order to maintaining the stable Ca^2+^ gradient in growing pollen tube. More precise research is required, however, to verify this hypothesis.

### Exchangeable Ca^2+^ dynamics during the progamic phase corresponds with extracellularly localized CRT in the nucellus and the synergids

One of our most interesting observations was the continuous location of CRT (before pollination and during the progamic phase) between nucellus cells surrounding the embryo sac and its preferential accumulation within the ER and the filiform apparatus of the synergids. Similar results were observed in *Petunia* and *Haemantus* ovules. Moreover, preferential localization of CRT in the filiform apparatus during the late progamic phase/fertilization was also confirmed in *Hyacinthus* ovule (Niedojadło et al. 2015). In contrast, we found that the level of exchangeable Ca^2+^ increased significantly during the progamic phase in *Petunia*; Ca^2+^ ppts strongly labeled the ER and dictyosomes as well as the intra/extracellular peripheries of both nucellus and synergid cells. An extremely high level of CRT was found within the filiform apparatus of the synergid cell. Similar results we found in *Haemanthus* micropylar pole of the ovule where a large mass of Ca^2+^ ppts was observed in the synergid after pollination. Since the probable function of CRT in the cellular peripheries of nucellus cells is similar to the function proposed in transmitting cells, the presence of CRT in the filiform apparatus needs an additional comment.

The filiform apparatus is a thickened cell wall of heterogeneous structure that involves fibrillar and amorphous fractions with numerous finger-like projections into the synergid cytoplasm (Płachno et al. 2014). This unique structure greatly increases the surface area of the plasma membrane at the micropylar pole of the synergid and is also associated with highly expanded ER. Several functions have been proposed for the filiform apparatus, including pollen tube guidance and reception, import of metabolites, and export of the pollen tube attractants, and numerous proteins have been found to localize in this specialized compartment (see review by Dresselhaus and Franklin-Tong 2013). We previously revealed a pollination-induced significant increase of CRT mRNA, CRT, and exchangeable Ca^2+^ levels in the synergid resulting in preferential accumulation of CRT and Ca^2+^ in the ER and the filiform apparatus (Lenartowski et al. 2014, 2015). This phenomenon is consistent with reports that, of all the cells in the embryo sac, the highest level of loosely bound Ca^2+^ is in the synergid (see review by Ge et al. 2007). It is still unknown whether Ca^2+^ is delivered from the extracellular space, the ER, or both. We postulate that CRT accumulated in the filiform apparatus may provide an exchangeable Ca^2+^ external store in the synergid and thus modulate the local concentration of cytoplasmic Ca^2+^ to prevent extra pollen tubes from entering the embryo sac. Generally, only one pollen tube enters each embryo sac in angiosperms, and precise regulation of the Ca^2+^ level in the penetrated ovule determines its receptivity. Upon pollen tube arrival at the *Arabidopsis* receptive synergid, Ca^2+^ oscillation begins at the micropylar pole and spreads toward the chalazal pole, and Ca^2+^ reaches a maximum level at pollen tube rupture Iwano et al. (2012). Even though recent studies demonstrate that pollen tube entry into the *Arabidopsis* synergid cell is observed at a site distinct from the filiform apparatus (Lesherm et al. 2013), it remains certain that this unique structure plays an important role in male and female gametophyte recognition. However, further studies are needed to understand the functional CRT accumulation within this highly specific structure during the progamic phase in angiosperms.

## Abbreviations

Ca^2+^: Calcium/calcium ions
Ca^2+^ ppts: Ca^2+^-antimonate precipitates
Cal MAb: Monoclonal antibody against callose
CRT: Calreticulin
CRT PAb: Polyclonal antibody against plant CRT
ER: Endoplasmic reticulum
